# Astragaloside IV ameliorates DSS-induced intestinal epithelial barrier dysfunction associated with modulation of tight junction proteins and MLCK/p-MLC and MyD88/TRAF6 signaling pathways

**DOI:** 10.3389/fphar.2026.1778530

**Published:** 2026-08-21

**Authors:** Mingyue Yang, Yang Wang, Wenxiu Jia, Zhiying Duan, Jing Wang, Xiaohui Huo, Shunjiang Xu, Yangyang Duan, Xiaolan Zhang

**Affiliations:** 1 Gastrointestinal Disease Diagnosis and Treatment Center, The First Hospital of Hebei Medical University, Shijiazhuang, China; 2 Department of Gastroenterology, Hebei Provincial Hospital of Chinese Medicine, Shijiazhuang, China; 3 Research Center, The First Hospital of Hebei Medical University, Shijiazhuang, China; 4 Department of Gastroenterology, The Second Hospital of Hebei Medical University, Shijiazhuang, China

**Keywords:** Astragaloside IV, inflammatory bowel disease, intestinal mucosal barrier, MLCK, MyD88/TRAF6, tight junction

## Abstract

**Objective:**

The integrity of the intestinal mucosal barrier is critical in the pathogenesis of Inflammatory Bowel Disease (IBD). While Astragaloside IV (ASIV) possesses potent anti-inflammatory properties, its specific effects on intestinal epithelial barrier function and the underlying mechanisms involving the Myosin Light Chain Kinase (MLCK) and Myeloid Differentiation Primary Response 88/TNF Receptor-Associated Factor 6 (MyD88/TRAF6) pathways in colitis remain elusive. This study aimed to investigate the protective effects of ASIV on tight junction (TJ) proteins and inflammatory signaling in dextran sulfate sodium (DSS)-induced colitis.

**Methods:**

A chronic colitis model was established in wild-type C57BL/6 mice using four cycles of DSS administration. ASIV (30 mg/kg/day) was administered orally starting from day 14. Disease activity index (DAI), colon length, and histopathological changes were assessed. Intestinal barrier function was evaluated by measuring bacterial translocation, colonic permeability to fluorescein isothiocyanate (FITC)-dextran, and serum lipopolysaccharide (LPS) levels. The expression of TJ proteins (ZO-1, Occludin, JAM-A, Claudin-2) and signaling molecules (MLCK, phosphorylated myosin light chain (p-MLC), MyD88, TRAF6) was determined by Western blot and quantitative real-time PCR (qRT-PCR). Representative immunofluorescence localization of Claudin-2 and ZO-1 in colonic tissues was included as supplementary qualitative evidence of tight junction remodeling.

**Results:**

ASIV treatment significantly attenuated DSS-induced weight loss, colon shortening, and histopathological damage (p < 0.05). Furthermore, ASIV markedly reduced colonic permeability, serum LPS levels, and bacterial translocation to mesenteric lymph nodes (p < 0.01). At the molecular level, ASIV reversed the DSS-induced downregulation of sealing TJs (ZO-1, Occludin, JAM-A) and suppressed the upregulation of the pore-forming protein Claudin-2 (p < 0.05). These protective effects were accompanied by significant inhibition of TNF-α mRNA expression, reduced MLCK protein expression, decreased MLC phosphorylation, and suppression of MyD88/TRAF6 signaling.

**Conclusion:**

ASIV effectively restores intestinal epithelial barrier integrity in experimental colitis. These protective effects are associated with rebalancing of TJ protein expression and localization and may be partially linked to attenuation of the TNF-α/MLCK/p-MLC and LPS/MyD88/TRAF6 signaling axes. These findings support ASIV as a promising barrier-protective candidate for mucosal homeostasis in IBD, while future cellular and pathway-intervention studies are needed to establish direct causality.

## Introduction

Inflammatory bowel disease (IBD), comprising Crohn’s disease (CD) and ulcerative colitis (UC), is a group of chronic, relapsing inflammatory disorders of the gastrointestinal tract (Huttenhower et al., 2014; Zhang et al., 2016). A pivotal factor in the pathogenesis of IBD is the compromise of the intestinal epithelial barrier, which leads to increased permeability and the subsequent translocation of luminal antigens and bacteria into the lamina propria (Martini et al., 2017; Yadav et al., 2016). This breach triggers an abnormal immune response characterized by the overproduction of pro-inflammatory cytokines, such as tumor necrosis factor-alpha (TNF-α), which further exacerbates barrier dysfunction, creating a vicious cycle of inflammation and tissue damage (Guo et al., 2017).

The intestinal barrier is maintained primarily by the apical junctional complex, of which tight junctions (TJs) are the most critical components. TJs consist of transmembrane proteins, including Occludin, Claudins, and Junctional Adhesion Molecules (JAMs), as well as cytosolic scaffold proteins like Zonula Occludens-1 (ZO-1) (Martini et al., 2017). Dysregulation of these proteins—specifically the downregulation of “sealing” proteins (ZO-1, Occludin, JAM-A) and the upregulation of “pore-forming” proteins (e.g., Claudin-2)—is a hallmark of IBD (Weber, 2012). Claudin-2 acts as a pore-forming tight junction protein, facilitating the paracellular leak of water and sodium (known as the “leak flux” diarrhea mechanism), which compromises barrier integrity and drives clinical diarrhea in colitis patients (Weber, 2012; Oami et al., 2024). Myosin light chain kinase (MLCK) plays a central role in the physiological regulation of TJ permeability; however, its overactivation by cytokines like TNF-α leads to the phosphorylation of the myosin light chain (p-MLC), causing cytoskeletal contraction and TJ disassembly (Su et al., 2013). Concurrently, the activation of the MyD88/TRAF6 signaling pathway by Toll-like receptors (TLRs) in response to bacterial lipopolysaccharide (LPS) is a key driver of the inflammatory cascade (Akira et al., 2001).

Currently approved conventional therapies for colitis include 5-aminosalicylates (5-ASA), systemic corticosteroids, immunomodulators, and biologic agents targeting TNF-α (e.g., infliximab) or integrins (e.g., vedolizumab) (Rain et al., 2022). However, these therapies are often restricted by significant clinical limitations, including corticosteroid dependency and systemic side effects, poor long-term remission with 5-ASA, and risk of opportunistic infections or secondary loss of response with biologics (Rain et al., 2022). Consequently, there is an urgent need to identify novel, safer, and multi-targeted therapeutic compounds—particularly natural product-derived agents—capable of promoting mucosal healing and resolving chronic inflammation. Traditional Chinese Medicine (TCM) offers a rich source of natural compounds with therapeutic potential for IBD (Guo et al., 2017). Astragalus membranaceus has been utilized for millennia for its immunomodulatory properties. Its major active saponin, Astragaloside IV (ASIV), exhibits diverse pharmacological activities, including anti-inflammatory, anti-oxidant, and anti-apoptotic effects (Zhang et al., 2020; Liu et al., 2011; Li et al., 2017). Recent studies indicate that ASIV can ameliorate experimental colitis by modulating macrophage polarization and inhibiting NF-κB signaling (Wu and Chen, 2019; Tian et al., 2021). Furthermore, emerging evidence suggests ASIV may protect the intestinal barrier via the PI3K/AKT pathway (Zhang et al., 2024). However, the precise mechanisms by which ASIV regulates specific TJ proteins, particularly the pore-forming Claudin-2, and its interaction with the MLCK and MyD88/TRAF6 pathways in chronic colitis remain to be fully elucidated. Therefore, examining whether ASIV is associated with coordinated changes in Claudin-2, sealing TJ proteins, and MLCK/p-MLC and MyD88/TRAF6 signaling may help clarify a barrier-centered mechanism underlying its protective effects in chronic colitis.

In the present study, we utilized a dextran sulfate sodium (DSS)-induced chronic colitis mouse model to evaluate the therapeutic efficacy of ASIV. We specifically adopted a hypothesis-driven strategy to investigate whether ASIV restores intestinal barrier integrity by rebalancing TJ protein expression—particularly by downregulating the pore-forming protein Claudin-2 and upregulating the sealing proteins ZO-1, Occludin, and JAM-A—and by attenuating the inflammatory and cytoskeletal signaling events represented by the TNF-α/MLCK/p-MLC and LPS/MyD88/TRAF6 axes. Because no unbiased transcriptomic screening was performed in the present study, these targets were selected based on established barrier biology and previous reports linking them to DSS-induced epithelial dysfunction rather than being identified through genome-wide screening.

## Materials and methods

### Animals and ethics statement

Female C57BL/6 mice (wild type, WT; age: 8–10 weeks; weight: 20–22 g) were obtained from the Animal Center of Hebei Medical University (Shijiazhuang, China). Female mice were selected for this study because they exhibit lower baseline aggressive behavior (such as fighting) in group-housing environments compared to male mice, which minimizes stress-induced confounding variations in baseline colitis severity, while remaining highly sensitive and reproducible in chronic DSS-induced mucosal injury models (Greuter et al., 2020). Mice were housed under specific pathogen-free (SPF) conditions with a 12-h light/dark cycle and *ad libitum* access to food and water (National Research Council, 2010). All animal experiments were approved by the Animal Care Committee of Hebei Medical University and conducted in strict accordance with the Chinese Health Guide for the Use of Laboratory Animals and followed the AVMA Guidelines for the Euthanasia of Animals (American Veterinary Medical Association, 2020).

### Reagents

Antibodies against ZO-1 (Cat# AF5145, 1:1000) were purchased from Santa Cruz Biotechnology (Dallas, TX, United States). Antibodies targeting JAM-A (Cat# CY5635, 1:1000), Claudin-2 (Cat# BY0158, 1:1000), Occludin (Cat# CY5997-20, 1:1000), TRAF6 (Cat# CY5174, 1:1000), MyD88 (Cat# AF5195-50ul, 1:1000), MLCK (Cat# CY5428-20ul, 1:1000), and p-MLC (Cat# EY2173, 1:1000) were obtained from Abcam (Cambridge, United Kingdom). Secondary HRP-conjugated antibodies (Cat# ZB-2301 and ZB-2305, 1:5000) were obtained from ZSGB-BIO (Beijing, China). RNasin was acquired from Beijing Dingguo Biotech Co. (Beijing, China). M-MLV reverse transcriptase was purchased from Invitrogen (Carlsbad, CA, United States). Primers for ZO-1, Occludin, JAM-A, Claudin-2, TNF-α, and GAPDH (Table 1) were synthesized by Beijing SBS Genetech Co. Ltd. (Beijing, China).

**TABLE 1:** Amplification primers for gene synthesis and qRT-PCR.

Gene	Amplification primers (5′ to 3′)	Product size (bp)
ZO-1	Sense: TGGTCTGTTTGCCCACTGTT Antisense: TCTGTACATGCTGGCCAAGG	150
Occludin	Sense: ACGTCCGACCCATGCTCTCT Antisense: AAGTCATCCGCAGGGGAGGT	147
JAM-A	Sense: GTGCCTACTCGGGCTTTTCT Antisense: AAGGTCACCCGGTCCTCATA	133
Claudin-2	Sense: ATCCTCTGCTTTTCCTGCCC Antisense: GAGCCTCTAGTTGCAAGGGG	135
TNF-α	Sense: CAGGCGGTGCCTATGTCTC Antisense: CGATCACCCCGAAGTTCAGTAG	112
GAPDH	Sense: TCGTCCCGTAGACAAAATGG Antisense: TTGAGGTCAATGAAGGGGTC	132

### Induction of colitis and treatment

Mice were randomly divided into three groups (n = 10 per group): Control, DSS, and DSS + ASIV. A chronic colitis model was established using a cyclic DSS administration protocol (Wirtz et al., 2017; Duan et al., 2022). This repeated 5-day DSS exposure followed by a 2-day recovery phase was selected to mimic relapsing-remitting chronic mucosal inflammation rather than acute epithelial injury, because repeated DSS cycles induce persistent clinical activity, crypt architectural distortion, inflammatory infiltration, colon shortening, and barrier dysfunction. Mice were subjected to four cycles of treatment; each cycle consisted of 2.5% (w/v) Dextran Sulfate Sodium (DSS) (molecular weight 36–50 kDa; MP Biomedicals, Solon, OH, United States; Lot# Q8247) dissolved in drinking water for 5 days, followed by distilled water for 2 days. The Control group received distilled water throughout the experimental period. In the DSS + ASIV group, ASIV (purity >98%; Shanghai Sigma-Aldrich, Shanghai, China, Cat# A1533) was suspended in 0.5% carboxymethyl cellulose (CMC) and administered by oral gavage once daily at a dose of 30 mg/kg/day starting from day 14 until sacrifice on day 29. Day 14 corresponded to the end of the second DSS cycle and was selected to evaluate the therapeutic, rather than prophylactic, effect of ASIV after colitis-related clinical manifestations had emerged but before terminal severe wasting occurred. The 30 mg/kg/day dose was selected based on previous ASIV-related experimental colitis and epithelial-barrier studies (Wu and Chen, 2019; Tian et al., 2021; Zhang et al., 2024) and our preliminary tolerability observations; no overt abnormal behavior, treatment-related mortality, or systemic toxicity was observed at this dose. Nevertheless, a complete dose-response and toxicity-ceiling analysis was not included in the present study and should be addressed in future studies. Detailed timeline cycles are depicted in Table 2. The DSS group received an equivalent volume of vehicle (0.5% CMC) during the same period. No animals died or were excluded during the modeling or treatment period; therefore, all 30 randomized mice (Control, n = 10; DSS, n = 10; DSS + ASIV, n = 10) were included in the final clinical, histological, permeability, and bacteriological analyses.

**TABLE 2:** Schematic timeline of the chronic DSS-induced colitis model and ASIV therapeutic regimen.

Phase/Cycle	Time interval (Days)	Drinking protocol	Gavage intervention (once daily)
Cycle 1	Days 1–5 Days 6–7	2.5% (w/v) DSS in water Distilled water only	None (no gavage)
Cycle 2	Days 8–12 Days 13–14	2.5% (w/v) DSS in water Distilled water only	ASIV treatment starts on day 14 (30 mg/kg/day, DSS + ASIV group)
Cycle 3	Days 15–19 Days 20–21	2.5% (w/v) DSS in water Distilled water only	Oral gavage of ASIV (DSS + ASIV) or 0.5% CMC vehicle (DSS group)
Cycle 4	Days 22–26 Days 27–28	2.5% (w/v) DSS in water Distilled water only	Oral gavage of ASIV (DSS + ASIV) or 0.5% CMC vehicle (DSS group)
Sacrifice	Day 29	Distilled water only	Euthanasia & sample collection

### Evaluation of disease activity index (DAI)

During the experimental period, body weight, stool consistency, and the presence of gross blood in feces were monitored daily. Fecal occult blood was assessed using a specific Fecal Occult Blood Test Kit (Nanjing Jiancheng Bioengineering Institute, Nanjing, China, Cat# C027-1-1). The Disease Activity Index (DAI) was calculated as the sum of scores for weight loss, stool consistency, and rectal bleeding, as previously described (Murthy et al., 1993; Leppkes and Neurath, 2020).

### Sample collection and histopathology

On day 29, mice were deeply anesthetized with 2% isoflurane (RWD Life Science, Shenzhen, China) and then humanely euthanized by cervical dislocation, in strict accordance with veterinary euthanasia guidelines (American Veterinary Medical Association, 2020). Blood samples were collected, centrifuged at 4 °C, and serum was stored at −80 °C for LPS analysis. The colon was excised, measured for length, and rinsed with ice-cold PBS. Distal colon segments (approx. 5 mm) were fixed in 4% paraformaldehyde, embedded in paraffin, and sectioned. Sections (4 μm) were stained with Hematoxylin and Eosin (H&E) for histopathological scoring using the Cooper HS scoring system (Cooper et al., 1993; Pavel et al., 2025). For each specimen, three non-consecutive sections were evaluated, and five random high-power fields (200×) were photographed using an Olympus BX53 microscope (Olympus, Tokyo, Japan) to record histological changes. Periodic Acid-Schiff (PAS) staining was performed to assess goblet cell depletion and mucin production (Cooper et al., 1993).

### Intestinal permeability and bacterial translocation

Intestinal barrier function was assessed *in vivo* using Fluorescein Isothiocyanate-Dextran (FITC-D) (Woting and Blaut, 2018; Wan et al., 2024). Mice were fasted for 4 h prior to sacrifice and administered FITC-D (60 mg/100 g body weight, MW 4 kDa; Sigma-Aldrich, St. Louis, MO, United States) by oral gavage. Serum fluorescence intensity was measured using a spectrophotometer (excitation: 490 nm; emission: 520 nm). Bacterial translocation was evaluated by culturing homogenates of mesenteric lymph nodes (MLN) on nutrient agar plates; colony-forming units (CFU) were counted after 24 h of incubation (Woting and Blaut, 2018). Serum LPS levels were quantified using an ELISA kit (Nanjing Jiancheng Bioengineering Institute, Cat# E012-1-1, detection range: 0.05-2.0 EU/mL) according to the manufacturer’s instructions.

### Transmission electron microscopy (TEM)

Colonic tissue samples (approx. 1 mm^3^) were fixed in 2.5% glutaraldehyde at 4 °C, post-fixed in 1% osmium tetroxide, and embedded in epoxy resin (Cooper et al., 1993). Ultrathin sections (60 nm) were stained with uranyl acetate and lead citrate and examined under a transmission electron microscope (H7650; Hitachi Ltd., Tokyo, Japan) to visualize ultrastructural changes in tight junctions. A qualitative assessment of TJ integrity was carried out based on the presence of continuous contact points and paracellular cleft expansion. Quantitative TEM morphometric analysis was not performed because the available retrospective imaging fields were not acquired using a standardized morphometric sampling protocol.

### Quantitative real-time PCR (qRT-PCR)

Total RNA was extracted from colonic tissues using TRIzol LS reagent. RNA concentration was determined spectrophotometrically. First-strand cDNA was synthesized using M-MLV reverse transcriptase (Tian et al., 2021). qRT-PCR was performed using the Opticon II system (MJ Research, Waltham, MA, United States) with SYBR Green. The relative mRNA expression levels were calculated using the 2^−ΔΔCt^ method, normalized to GAPDH. Primer sequences are listed in Table 1.

### Representative immunofluorescence localization of tight junction proteins

To provide additional tissue-localization support for tight junction remodeling, representative immunofluorescence images of Claudin-2 and ZO-1 were prepared from distal colon tissue sections from the Control, DSS, and DSS + ASIV groups. Nuclei were counterstained with 4,6-diamidino-2-phenylindole (DAPI), and images were compared across groups under matched acquisition settings. This supplemental experiment was used as qualitative tissue-localization evidence and was interpreted together with the qRT-PCR and Western blot results, rather than as an independent quantitative endpoint. Briefly, distal colon sections were subjected to antigen retrieval, blocked with normal serum, and incubated overnight at 4 °C with primary antibodies against Claudin-2 and ZO-1. After washing, sections were incubated with fluorophore-conjugated secondary antibodies and counterstained with DAPI. Images were acquired under identical exposure settings for each marker. Representative images were selected from n = 3 mice per group, with at least three non-overlapping fields examined per mouse.

### Western blotting

Colonic tissues were homogenized in lysis buffer, and protein concentrations were determined using the BCA assay (Tian et al., 2021). Equal amounts of protein (30 μg) were separated by 10% SDS-PAGE and transferred to PVDF membranes (Millipore, Burlington, MA, United States). Membranes were blocked with 5% non-fat milk and incubated overnight at 4 °C with primary antibodies against ZO-1, Occludin, JAM-A, Claudin-2, MLCK, p-MLC, MyD88, TRAF6, and β-actin. After incubation with secondary antibodies, bands were visualized using ECL chemiluminescence and analyzed with Image Pro-Plus 6.0 software. β-actin served as the loading control.

### Statistical analysis

Statistical analyses were performed using SPSS 18.0 software (SPSS Inc., Chicago, IL, United States), and figures were redrawn using GraphPad Prism software (version 10.0; GraphPad Software, Boston, MA, United States). Continuous variables are presented as Mean ± Standard Deviation (SD). The sample size of n = 10 mice per group was selected according to published chronic DSS colitis protocols and our previous modeling experience, which indicated that this number was sufficient to detect biologically meaningful differences in DAI, colon length, histological injury, and permeability-related endpoints. Animal husbandry, clinical assessments, and histological evaluations were performed by researchers blinded to the experimental group allocations to eliminate observer bias. Statistical outliers were assessed using Grubbs’ test with an alpha level of 0.05; no statistical outliers were identified, and thus all animal data were included in the final analysis. Differences among groups were analyzed using one-way Analysis of Variance (ANOVA) followed by the Student-Newman-Keuls (SNK) *post hoc* test. A p-value <0.05 was considered statistically significant.

## Results

### ASIV alleviates clinical symptoms and colonic injury in DSS-induced colitis

**FIGURE 1:**
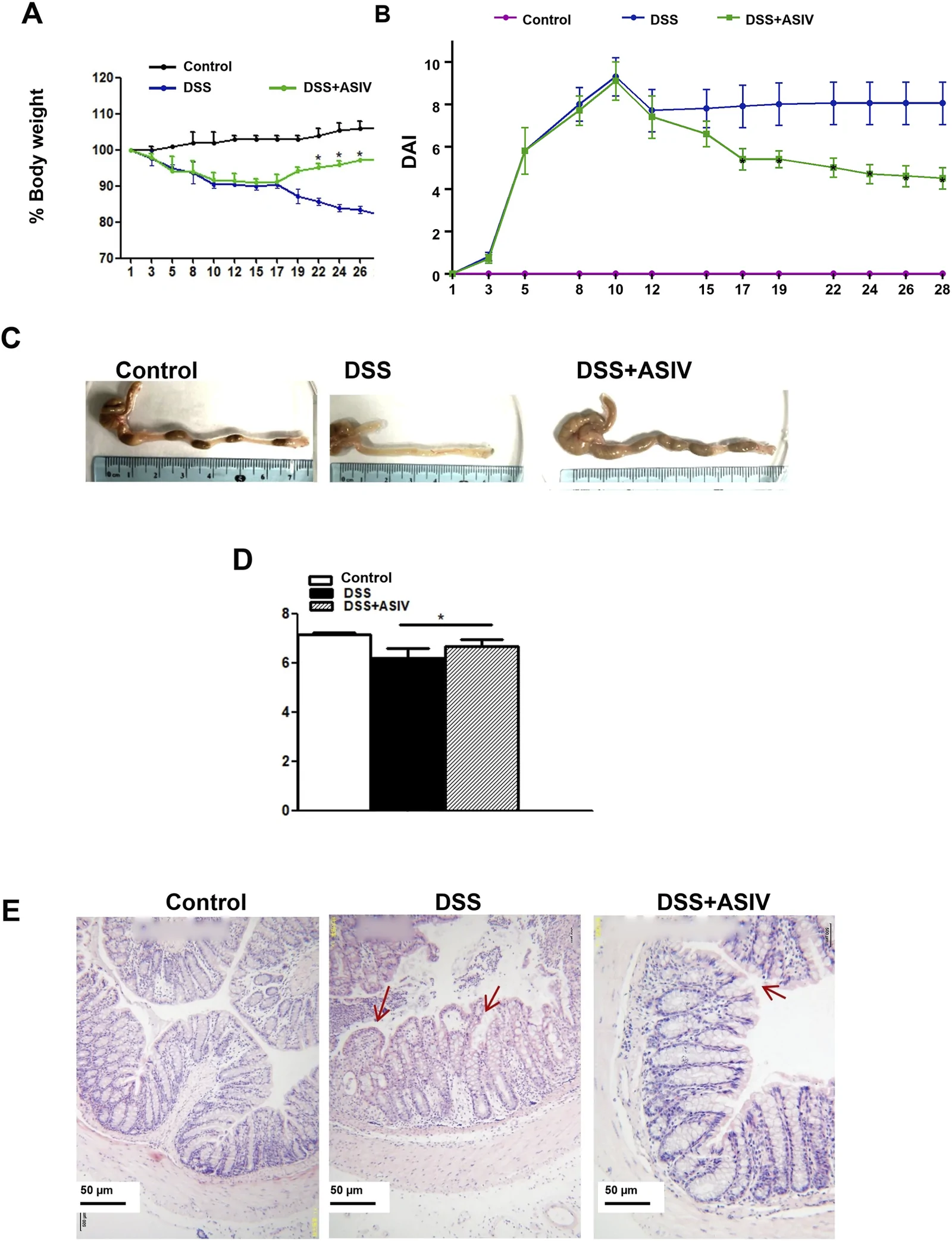
Effects of Astragaloside IV (ASIV) on body weight, Disease Activity Index (DAI), and histopathology in Dextran Sulfate Sodium (DSS)-induced colitis. **(A,B)** Changes in percentage body weight and DAI over the experimental period. DSS induction caused significant weight loss and elevated DAI, which were ameliorated by ASIV. **(C,D)** Representative images of the colon and quantification of colon length. ASIV treatment prevented DSS-induced colon shortening. **(E)** Representative Hematoxylin and Eosin (H&E) staining (200×; scale bar = 50 μm). Red arrows and circles indicate mucosal erosion, edema, and inflammatory cell infiltration. Data are presented as Mean ± SD (n = 10 mice/group). *p < 0.05 vs. DSS. Individual animal-level data were included in the analysis for all *in vivo* endpoints (n = 10 mice/group); no animal died or was excluded during the experiment.

Successful establishment of chronic DSS-induced colitis was confirmed by progressive body-weight loss, elevated DAI scores, diarrhea/rectal bleeding, colon shortening, and histopathological injury, including epithelial erosion, crypt architectural distortion, edema, and inflammatory-cell infiltration. These persistent clinical and histological changes after repeated DSS cycles supported successful generation of a chronic inflammatory model rather than a transient acute injury model. Compared with the Control group, the DSS group exhibited a significantly increased DAI score and reduced body weight, with the nadir of weight loss occurring on day 28 (Figures 1A,B). Treatment with ASIV significantly mitigated these symptoms, resulting in lower DAI scores and improved body weight maintenance compared to the untreated DSS group (p < 0.05). Macroscopically, DSS induction caused significant colon shortening, which was partially prevented by ASIV treatment (Figures 1C,D). Histopathological analysis (H&E) revealed severe epithelial disruption, crypt distortion, edema, and inflammatory cell infiltration in the DSS group. In contrast, the DSS + ASIV group showed preserved epithelial architecture and significantly lower Cooper HS scores (Table 3; Figure 1E).

**TABLE 3:** Histological scoring of colon tissues based on the Cooper HS system.

Group	Cooper HS score (Mean ± SD)
Control	0.33 ± 0.25
DSS	8.83 ± 1.17^a^
DSS + ASIV	5.83 ± 0.75^b^

Data are expressed as Mean ± SD (n = 10 mice per group).

^a^ p < 0.05 vs. Control.

^b^ p < 0.05 vs. DSS, group. Pathological scoring criteria are detailed in Cooper et al. (1993) (Cooper HS, Murthy SN, Shah RS, Sedergran DJ: Clinicopathologic study of dextran sulfate sodium experimental murine colitis. Lab Invest 1993, 69 (2):238-249).

### ASIV preserves mucosal ultrastructure and goblet cell-associated mucus barrier

**FIGURE 2:**
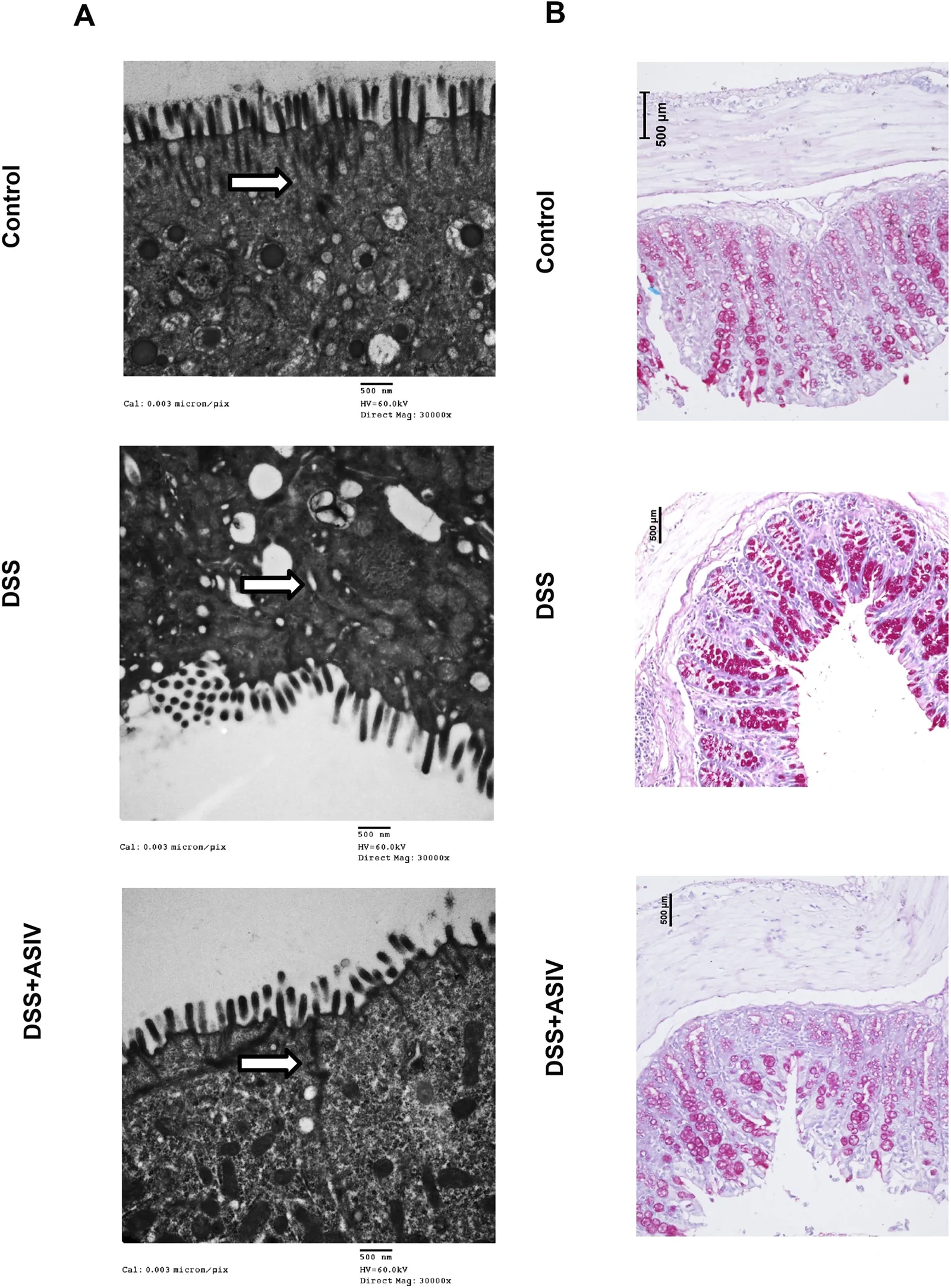
Structural changes evaluated by Transmission Electron Microscopy (TEM) and Periodic Acid-Schiff (PAS) staining. **(A)** Representative TEM images of the colonic mucosal tight junctions (magnification ×30000; scale bar = 500 nm). White arrows point to tight junction complexes. **(B)** PAS staining showing colonic goblet cells (200×; scale bar = 500 μm). Mucin depletion is evident in the DSS group and restored by ASIV. Data represent *n* = 10 mice/group. All representative images were selected from the same experimental groups for which n = 10 mice/group were included in the final analysis.

To further assess mucosal integrity, we performed PAS staining and TEM. PAS staining demonstrated a marked reduction in PAS-positive goblet cells and mucin staining in the DSS group. ASIV treatment partially restored PAS-positive goblet cell abundance and mucin staining intensity (Figure 2B), suggesting improvement of the mucus barrier. However, whether ASIV directly regulates goblet cell differentiation was not determined in the present study and requires further analysis using mucus- and goblet-cell-associated markers such as MUC2, KLF4, SPDEF, or ATOH1. Under TEM, the Control group displayed intact epithelial TJs and microvilli. The DSS group exhibited widened paracellular spaces and disrupted TJs. Consistent with the histological findings, ASIV treatment ameliorated these ultrastructural defects, maintaining the integrity of the epithelial barrier (Figure 2A).

### ASIV reduces intestinal permeability and bacterial translocation

**FIGURE 3:**
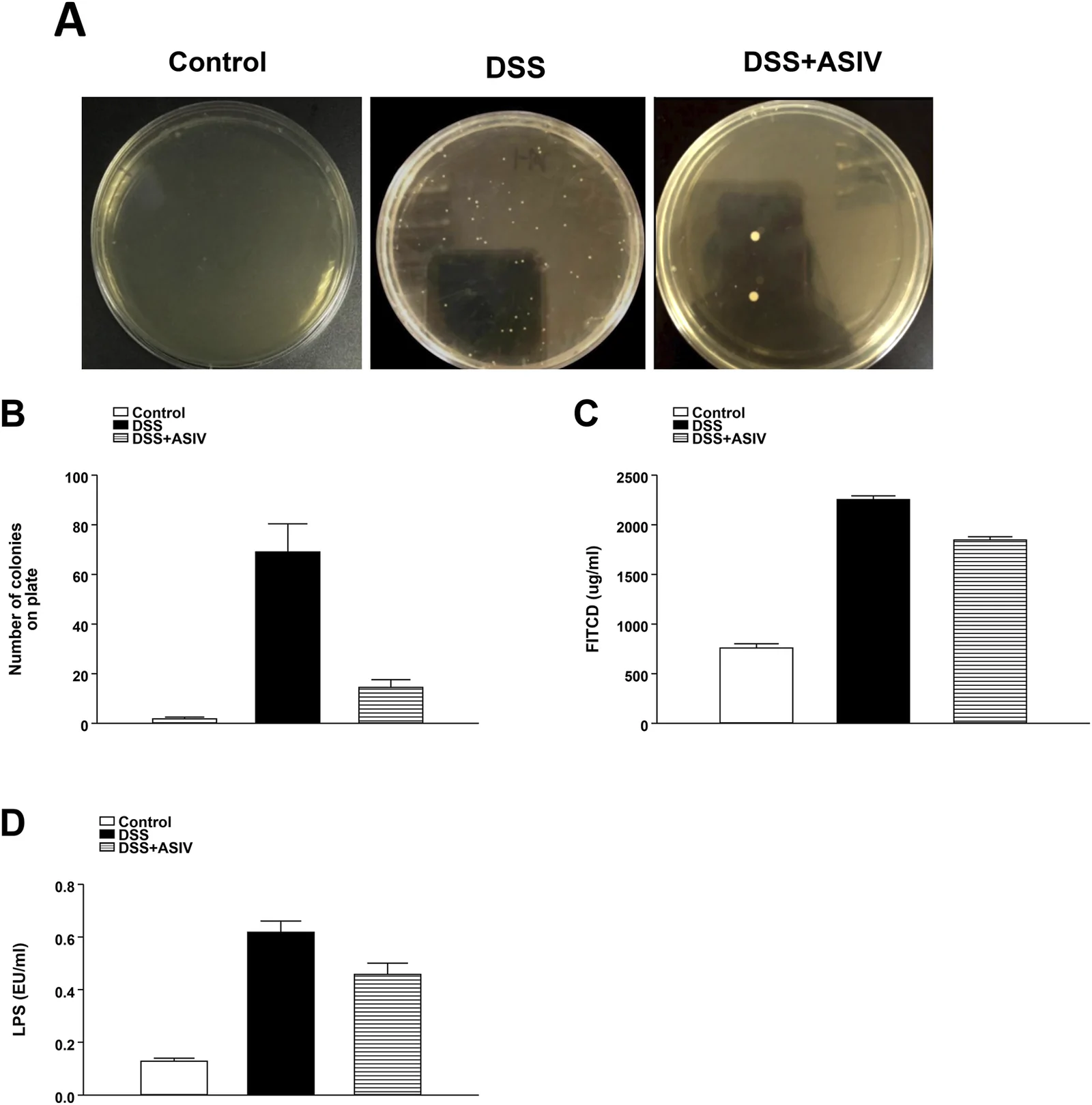
Effects of ASIV on intestinal permeability and bacterial translocation. **(A,B)** Bacterial colonies cultured from Mesenteric Lymph Node (MLN) homogenates. ASIV significantly reduced bacterial translocation compared to the DSS group. **(C)** Intestinal permeability assessed by serum FITC-dextran concentration. **(D)** Serum lipopolysaccharide (LPS) levels. Data are presented as Mean ± SD (n = 10 mice/group). *p < 0.01 vs. DSS. In bar graphs, open, solid black, and hatched bars indicate Control, DSS, and DSS + ASIV, respectively. All *in vivo* permeability and bacterial-translocation analyses included n = 10 mice/group, with no post-randomization exclusion.

Functional barrier defects were assessed by measuring FITC-D permeability, bacterial translocation, and serum LPS. The DSS group showed a dramatic increase in serum FITC-D concentration compared to controls (2,256.7 ± 32.7 μg/mL vs. 763.3 ± 36.1 μg/mL, p < 0.01), indicating severe barrier leakage. ASIV treatment significantly reduced FITC-D permeability (1851.7 ± 26.4 μg/mL, p < 0.01) (Figure 3C). Similarly, bacterial translocation to MLNs was significantly higher in the DSS group but was markedly suppressed by ASIV (14.67 ± 2.88 vs. 69.17 ± 11.14 CFU, p < 0.01) (Figures 3A,B). Serum LPS levels followed the same trend, being significantly elevated in the model group and reduced upon ASIV administration (Figure 3D). These findings indicate that ASIV reduced functional leakage and bacterial passage across the damaged intestinal barrier; however, they do not demonstrate a direct antibacterial effect of ASIV.

### ASIV regulates the expression and tissue localization of tight junction proteins

**FIGURE 4:**
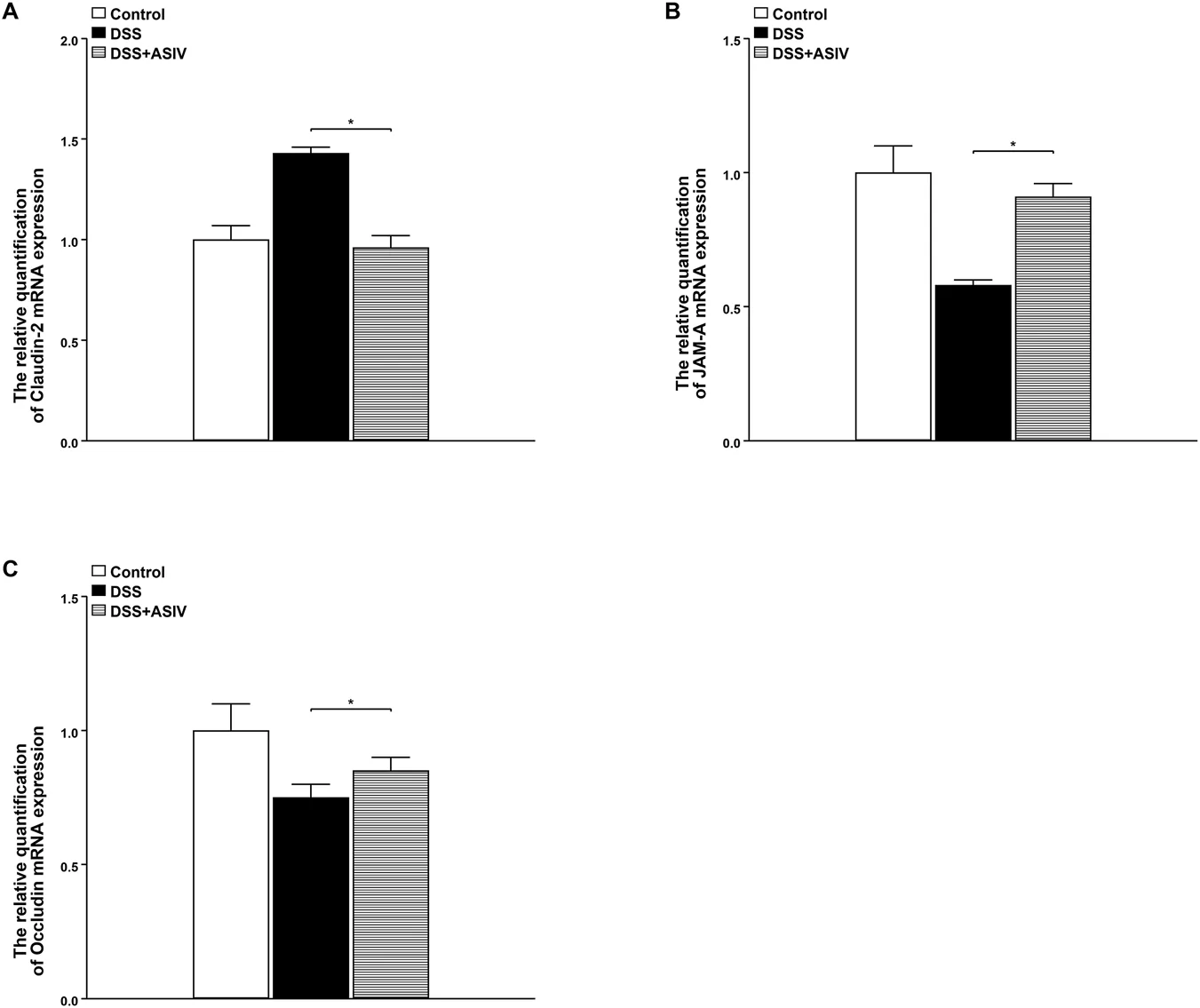
mRNA expression of Tight Junction proteins in colonic tissue. Relative colonic mRNA levels of Occludin **(B)**, JAM-A **(C)**, and Claudin-2 **(A)** determined by qRT-PCR. DSS treatment decreased Occludin and JAM-A while increasing Claudin-2. ASIV treatment significantly reversed these trends. Data are presented as Mean ± SD (n = 3 independent molecular experiments). *p < 0.05 vs. DSS. In bar graphs, open, solid black, and hatched bars indicate Control, DSS, and DSS + ASIV, respectively.

**FIGURE 5:**
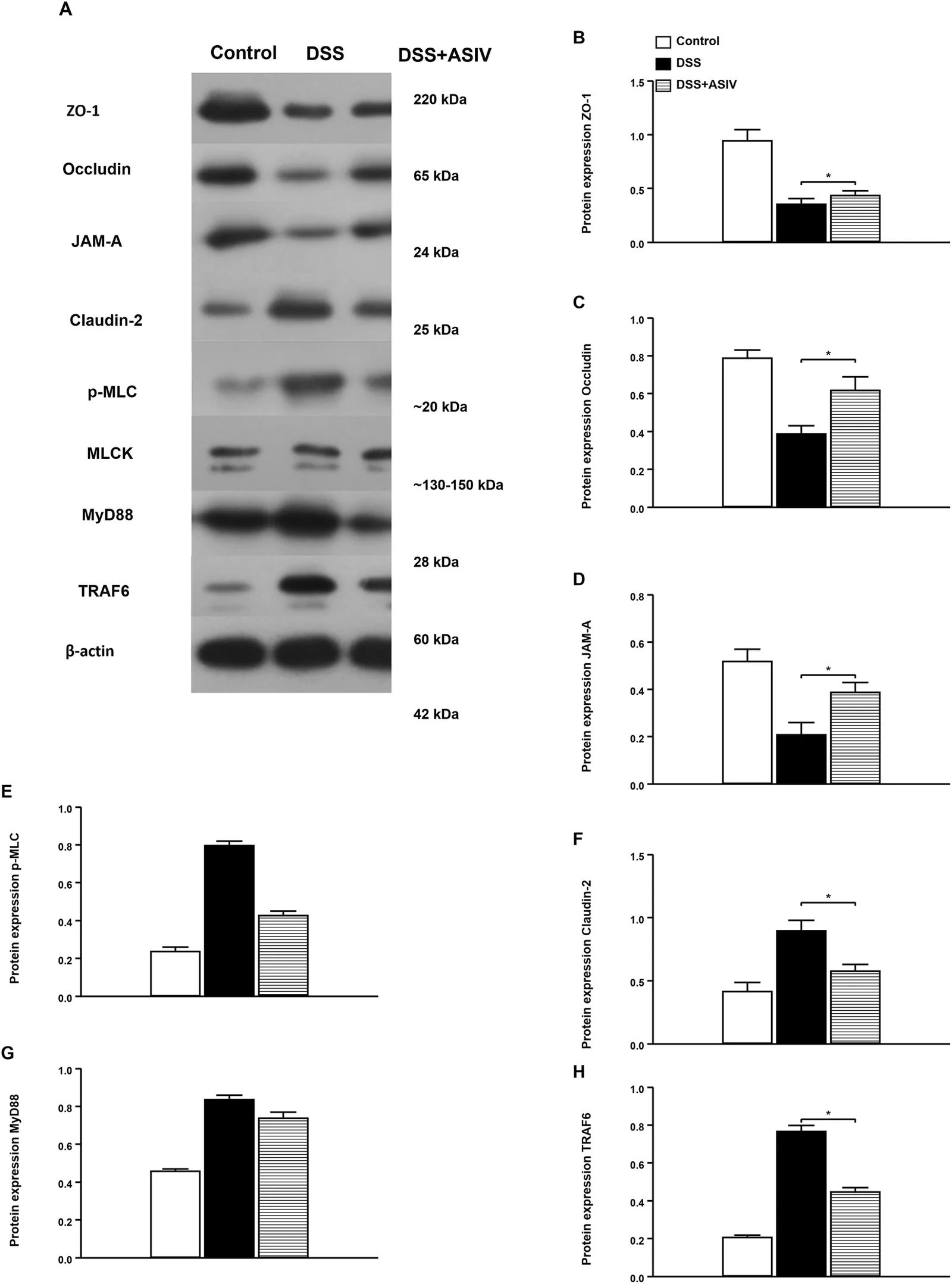
Protein expression of Tight Junctions and Inflammatory Signaling molecules. **(A)** Western blot bands for ZO-1, Occludin, JAM-A, Claudin-2, p-MLC, MLCK, MyD88, TRAF6, and β-actin in colonic lysates. Molecular weights are indicated next to the corresponding bands, and full-length original blots with molecular weight ladders and matched β-actin loading controls are provided in the Supplementary Material. **(B–H)** Densitometric analysis of target protein expression normalized to β-actin. Because total MLC was not separately detected, p-MLC immunoreactivity was normalized to β-actin and interpreted as a relative indicator of MLC phosphorylation-associated contractile activation. Compared with the DSS group, the ASIV group showed higher levels of ZO-1, Occludin, and JAM-A, and lower levels of Claudin-2, p-MLC, MLCK, MyD88, and TRAF6. Data are presented as Mean ± SD (n = 3 independent molecular experiments). *p < 0.05 vs. DSS. In bar graphs, open, solid black, and hatched bars indicate Control, DSS, and DSS + ASIV, respectively. Western blot molecular-weight annotations are presented uniformly in kDa beside the corresponding bands: ZO-1, 220 kDa; Occludin, 65 kDa; JAM-A, 24 kDa; Claudin-2, 25 kDa; p-MLC, approximately 20 kDa; epithelial low-molecular-weight MLCK, approximately 130–150 kDa; MyD88, 28 kDa; TRAF6, 60 kDa; and β-actin, 42 kDa.

Because TJ proteins are established structural determinants of epithelial paracellular permeability, we examined representative sealing proteins (ZO-1, Occludin, and JAM-A) and the pore-forming protein Claudin-2 to explore the molecular basis of ASIV-associated barrier protection. Western blot and qRT-PCR analyses revealed that DSS treatment significantly downregulated the expression of ZO-1, Occludin, and JAM-A, while significantly upregulating Claudin-2 compared to controls (p < 0.05). ASIV treatment reversed this dysregulation, significantly increasing the levels of ZO-1, Occludin, and JAM-A, and suppressing Claudin-2 expression compared to the DSS group (p < 0.05) (Figures 4, 5A–H). These results support an association between ASIV-mediated barrier improvement and rebalancing of TJ protein composition.

To provide additional tissue-localization evidence, representative immunofluorescence staining of Claudin-2 and ZO-1 was added as Supplementary Figure S1. DSS-induced colitis was accompanied by enhanced or more diffuse Claudin-2 immunofluorescence and weakened, discontinuous ZO-1 localization in the epithelial region. ASIV treatment partially restored the junctional pattern of ZO-1 and reduced the abnormal Claudin-2 staining pattern. These qualitative localization findings were consistent with the qRT-PCR and Western blot results, supporting an association between ASIV-mediated barrier protection and rebalancing of tight junction composition and localization. Because these immunofluorescence images were intended to provide supplemental localization evidence, they were interpreted qualitatively and were not used as independent quantitative endpoints. Further quantitative co-localization with epithelial, goblet-cell, and immune-cell markers will be required to determine the precise cellular compartment responsible for these changes.

### ASIV modulates TNF-α, MLCK/p-MLC, and MyD88/TRAF6 signaling pathways

**FIGURE 6:**
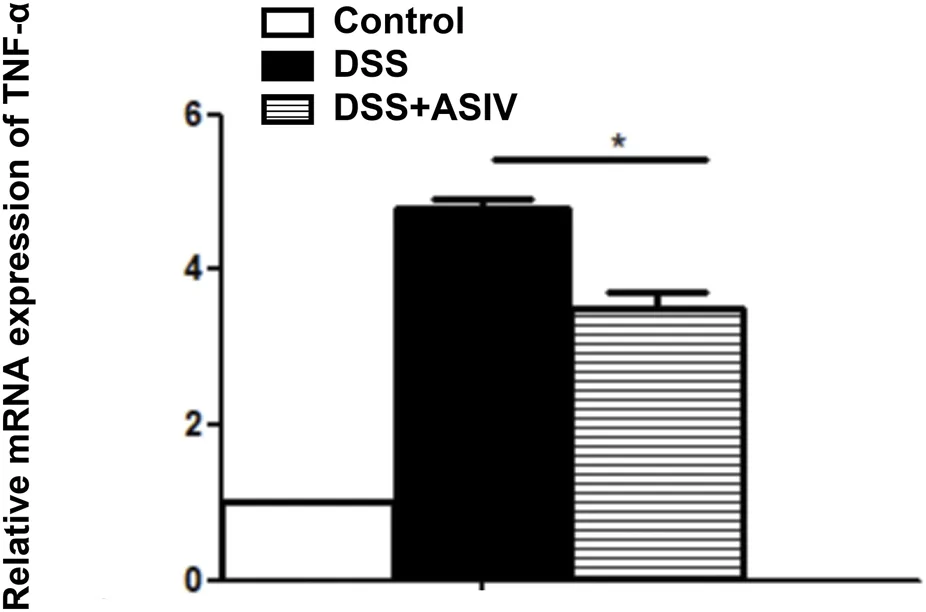
Relative colonic mRNA expression of TNF-α. TNF-α transcript levels were significantly upregulated in the DSS group compared to controls and significantly downregulated by ASIV treatment. Data are presented as Mean ± SD (n = 3 independent experiments). *p < 0.05 vs. DSS. In bar graphs, open, solid black, and hatched bars indicate Control, DSS, and DSS + ASIV, respectively.

**FIGURE 7:**
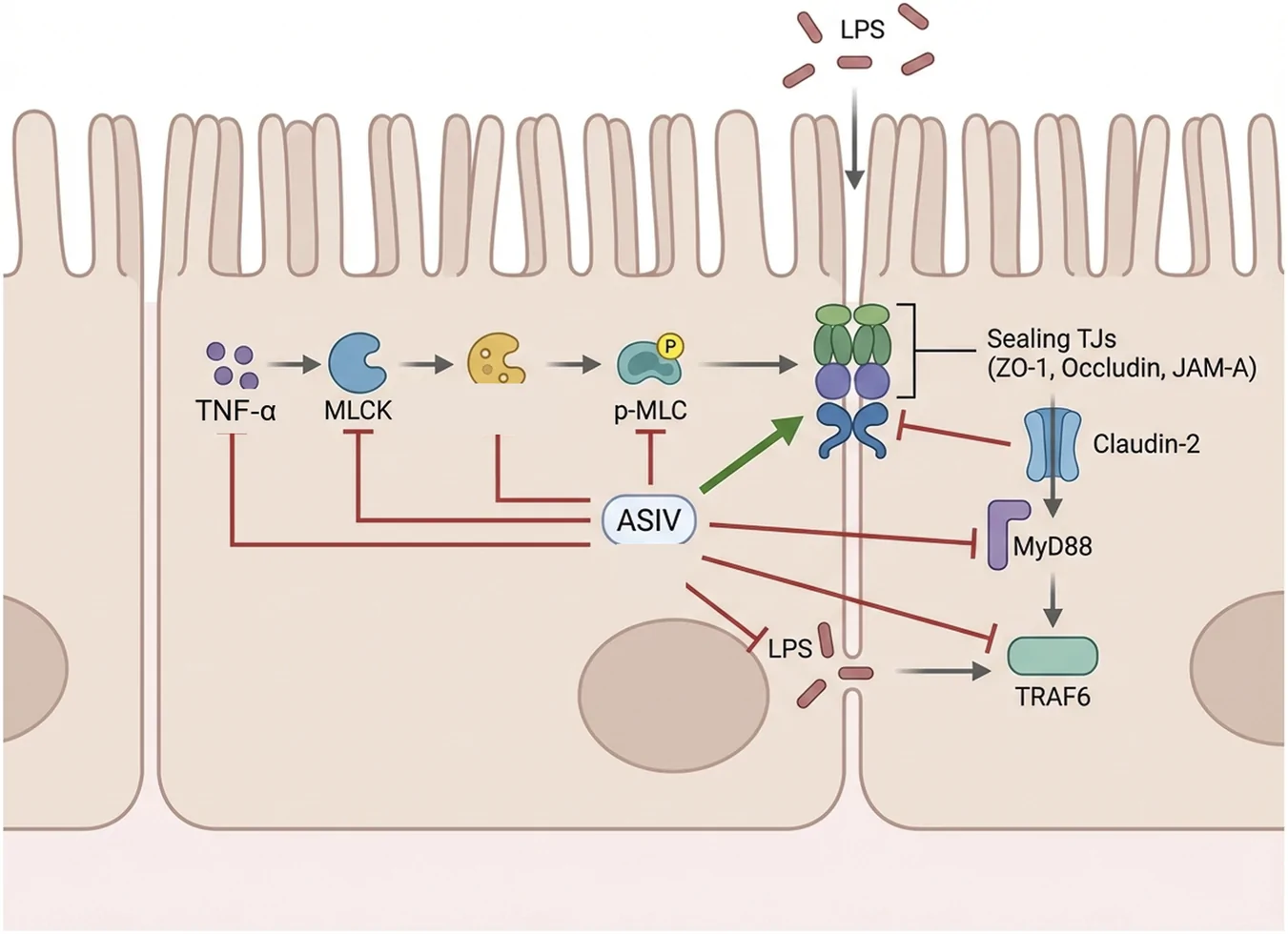
Schematic illustration of the proposed ASIV-associated barrier-protective mechanism. Luminal LPS and pro-inflammatory cytokines (e.g., TNF-α) are associated with barrier disruption. TNF-α activates the MLCK/p-MLC signaling pathway, leading to disassembly of sealing tight junctions (ZO-1, Occludin, JAM-A) and upregulation of pore-forming Claudin-2. Concurrently, LPS-associated MyD88/TRAF6 signaling amplifies inflammatory responses. ASIV treatment was associated with restoration of sealing tight junction proteins, suppression of Claudin-2, reduced permeability, and attenuation of MLCK/p-MLC and MyD88/TRAF6 signaling. This model remains hypothesis-generating and requires validation by cellular and pathway-intervention studies.

To further interpret the upstream signaling context of TJ remodeling, we assessed selected inflammatory and cytoskeletal signaling molecules based on their established links with DSS-induced barrier dysfunction. The mRNA expression of the pro-inflammatory cytokine TNF-α was significantly upregulated in the DSS group and suppressed by ASIV (Figure 6). Furthermore, DSS treatment led to a significant increase in MLCK protein expression and MLC phosphorylation (p-MLC), as well as increased expression of MyD88 and TRAF6. The primary antibody for MLCK utilized in this study targeted the epithelial-specific low-molecular-weight MLCK isoform (∼130–150 kDa), which was strongly induced by DSS and suppressed by ASIV. Notably, the immunoblot for MLC presented double bands corresponding to the non-phosphorylated (∼18 kDa) and phosphorylated states (∼20 kDa) of MLC; we specifically quantified the upper band corresponding to the phosphorylated state (p-MLC, ∼20 kDa) associated with cytoskeletal contraction. ASIV treatment significantly inhibited the upregulation of MLCK, p-MLC, MyD88, and TRAF6 (p < 0.05) (Figure 5). These data indicate that ASIV-associated barrier restoration occurs together with suppression of MLCK/p-MLC and MyD88/TRAF6 signaling, although causal dependence on these pathways remains to be validated by inhibitor, rescue, or genetic-intervention experiments. A schematic summary of the proposed ASIV-associated barrier-protective mechanism is shown in Figure 7.

## Discussion

The intestinal epithelial barrier serves as the first line of defense against luminal pathogens. Its disruption is a pivotal event in the onset and perpetuation of IBD (Martini et al., 2017; Weber, 2012; Mehandru and Colombel, 2021). In this study, we demonstrated that ASIV, a major bioactive saponin from Astragalus membranaceus, effectively ameliorates DSS-induced chronic colitis. The therapeutic efficacy of ASIV was evidenced by reduced DAI scores, preserved colon length, improved histopathology, decreased FITC-dextran permeability, reduced serum LPS, and diminished bacterial translocation. Importantly, the present study should be interpreted as an *in vivo* tissue-level investigation demonstrating strong associations between ASIV-mediated barrier protection and selected TJ/inflammatory signaling changes, rather than definitive proof of direct epithelial-cell-autonomous mechanisms.

Structurally, the intestinal barrier’s paracellular permeability is defined by tight junction proteins. The targets examined in this study were selected through a hypothesis-driven approach based on established barrier biology rather than through transcriptomic or proteomic screening. Claudin-2 was prioritized because it forms cation-selective pores that increase epithelial permeability to water and small cations (Oami et al., 2024; Raju et al., 2020). Upregulation of Claudin-2 is consistently observed in IBD patients and is considered a critical driver of “leak flux” diarrhea (Raju et al., 2020; Luettig et al., 2015). In contrast, ZO-1, Occludin, and JAM-A represent important sealing/scaffolding components required for epithelial junctional integrity. Our data showed that ASIV significantly suppressed DSS-induced Claudin-2 overexpression while restoring ZO-1, Occludin, and JAM-A. This coordinated rebalancing of pore-forming and sealing TJ components provides a plausible structural explanation for the observed reduction in FITC-dextran permeability, serum LPS, and bacterial translocation.

Moving upstream to the intracellular signaling drivers, we explored signaling pathways that are known to coordinate cytokine-induced TJ disassembly. TNF-α is a master regulator of intestinal inflammation and is known to increase barrier permeability by upregulating MLCK expression and activity (Su et al., 2013; Tao et al., 2020; Huang et al., 2020). Activated MLCK phosphorylates the myosin light chain (p-MLC), inducing contraction of the perijunctional actomyosin ring and opening the paracellular pathway. Our results demonstrate that ASIV treatment concurrently reduced TNF-α mRNA levels and inhibited MLCK/p-MLC activation. Thus, ASIV-associated barrier protection may be partially linked to dampening of the TNF-α/MLCK/p-MLC axis. However, because no MLCK activator, MLCK inhibitor, or genetic rescue experiment was included, these data should be considered correlative rather than causal.

In addition to cytoskeletal regulation, extracellular inflammatory amplification plays an important role in colitis-associated barrier damage. We observed reduced expression of MyD88 and TRAF6 in ASIV-treated mice. MyD88 is a canonical adaptor protein for TLRs (except TLR3) and IL-1 receptors. Upon activation by LPS or cytokines (e.g., IL-1β), MyD88 recruits TRAF6, leading to NF-κB activation and the production of inflammatory cytokines (Akira et al., 2001; Duan et al., 2022; Zhang and Frei, 2015). The reduction in serum LPS and bacterial translocation observed in our study suggests reinforcement of the barrier, which may in turn reduce the luminal TLR ligands available to stimulate this pathway. Conversely, suppression of MyD88/TRAF6 signaling may contribute to interruption of the inflammatory feedback loop. Nevertheless, the current tissue-level data cannot distinguish whether ASIV acts directly on epithelial cells, indirectly through immune-cell modulation, or through combined effects. Future tissue-localization or epithelial-cell-specific experiments are needed to clarify the cell type responsible for these changes. While we focused on TNF-α, it is acknowledged that other cytokines such as IL-1β and IL-6 also play significant roles in IBD pathogenesis (Leppkes and Neurath, 2020; Pavel et al., 2025). Although direct measurements of IL-1β and IL-6 were not performed in our animal model due to serum volume constraints, recent studies suggest ASIV also modulates these broader cytokine networks (Tian et al., 2021; Wan et al., 2024).

Pharmacokinetically, ASIV is recognized to have relatively low oral bioavailability (typically <2.5% in rodents) due to its high molecular weight, poor aqueous solubility, and susceptibility to active efflux by P-glycoprotein in the gut (Yang et al., 2022). Despite this low systemic bioavailability, oral administration of ASIV exerts prominent local therapeutic effects on the intestinal tract, as it directly bathes the mucosal lining at high local concentrations, facilitating direct contact with epithelial cells to restore TJ expression and suppress mucosal inflammation. To overcome pharmacokinetic limitations and translate these preclinical findings into human clinical trials, advanced drug delivery systems, such as lipid nanoparticles, biomimetic nanocarriers, or gut-targeted microcapsules, are actively being developed to improve ASIV absorption and systemic exposure (Yang et al., 2022). Clinically, while *Astragalus membranaceus* extracts containing ASIV have a historically favorable safety profile with minimal adverse events and an absence of severe drug-drug interactions, systematic reviews emphasize that conclusive human clinical trials are still required to establish the ideal therapeutic dose and long-term safety profile of purified ASIV, a necessary step to fully support its translational potential as a safe adjunctive therapy for colitis patients (Peng et al., 2023).

Despite these promising findings, our study has limitations. First, our mechanistic analysis relied primarily on tissue-level protein and mRNA expression, and direct causal evidence using pathway inhibitors, activators, rescue experiments, or knockout models was not available. Second, although representative immunofluorescence localization images were added in this revision, we did not perform *in vitro* epithelial-cell experiments or quantitative co-localization using epithelial markers (e.g., EpCAM/Villin), goblet-cell markers (e.g., MUC2), or immune-cell markers (e.g., CD45/F4/80). Therefore, the present data cannot determine whether ASIV acts directly on intestinal epithelial cells or indirectly through immune-cell regulation. Third, we did not perform transcriptomic or proteomic screening; therefore, the selected TJ and signaling targets were hypothesis-driven rather than unbiased discovery targets. Fourth, only a single ASIV dose was examined, and a complete dose-response relationship and toxicity ceiling were not established. Finally, quantitative TEM morphometry and additional goblet-cell differentiation markers were not included. These limitations have been explicitly acknowledged, and the mechanistic conclusions have been revised to avoid overstating causality. We agree that additional *in vitro* validation using intestinal epithelial cell lines (such as Caco-2 or NCM460), macrophage models, and epithelial-immune co-culture systems would further strengthen the mechanistic interpretation of the MLCK/p-MLC and MyD88/TRAF6 pathways; these experiments have therefore been specified as a priority for the next stage of this project.

## Future perspectives

To definitively translate these findings into advanced therapeutics, our future studies will employ: (1) *in vitro* intestinal epithelial-cell models such as Caco-2, NCM460, or intestinal organoids challenged with TNF-α and/or LPS to determine whether ASIV directly improves TEER, FITC-dextran flux, and TJ localization; (2) quantitative tissue-localization approaches, including co-staining of TJ proteins with epithelial markers (e.g., EpCAM/Villin), goblet-cell or mucus markers (e.g., MUC2), and immune-cell markers (e.g., CD45 or F4/80), to distinguish epithelial and immune compartments; (3) genetic knockout models or pathway-intervention strategies, such as MyD88−/− mice, epithelial-specific MLCK−/− mice, MLCK inhibitors (e.g., ML-7), or rescue experiments, to establish whether the protective effects of ASIV are abolished when these pathways are manipulated; and (4) development of colon-targeted nanoparticle or microcapsule formulations of ASIV to enhance local mucosal bioavailability and evaluate clinical dose-response characteristics in randomized controlled trials.

## Conclusion

In conclusion, ASIV alleviates DSS-induced chronic colitis and restores intestinal epithelial barrier integrity. These protective effects are associated with rebalancing of tight junction composition and localization, particularly suppression of pore-forming Claudin-2 and restoration of sealing proteins such as ZO-1, Occludin, and JAM-A, and with attenuation of MLCK/p-MLC and MyD88/TRAF6 signaling. Further cellular, quantitative co-localization, and pathway-intervention studies are required to confirm whether these signaling changes are direct causal mechanisms of ASIV-mediated barrier protection. ASIV remains a potential therapeutic candidate for targeting mucosal healing in IBD.

## Data Availability

The original contributions presented in the study are included in the article/Supplementary Material, further inquiries can be directed to the corresponding author.

## Supplementary material

SUPPLEMENTARY FIGURE S1 Representative immunofluorescence localization of Claudin-2 and ZO-1 in colonic tissue sections. Claudin-2 and ZO-1 staining was compared across Control, DSS, and DSS + ASIV groups, with DAPI counterstaining of nuclei. Original magnification, ×200; scale bar = 50 µm. The images provide qualitative tissue-localization support for the qRT-PCR and Western blot findings and were not used as an independent quantitative endpoint. Representative images were selected from n = 3 mice per group, with at least three non-overlapping fields examined per mouse.
