# Supplementary material for: Astragaloside IV ameliorates DSS-induced intestinal epithelial barrier dysfunction associated with modulation of tight junction proteins and MLCK/p-MLC and MyD88/TRAF6 signaling pathways
Source: Front Pharmacol. 2026 Aug 21;17:1778530. doi: 10.3389/fphar.2026.1778530 (PMC13541548; doi:10.3389/fphar.2026.1778530)
Supplement: Supplementary file 1 [file Supplementaryfile1.docx]

ZO-1

Occludin

JAM-A

Claudin-2

p-MLC

MLCK

MyD88

TRAF6

β-actin
